# A γA-Crystallin Mouse Mutant *Secc *with Small Eye, Cataract and Closed Eyelid

**DOI:** 10.1371/journal.pone.0160691

**Published:** 2016-08-11

**Authors:** Man Hei Cheng, Chung Nga Tam, Kwong Wai Choy, Wai Hung Tsang, Sze Lan Tsang, Chi Pui Pang, You Qiang Song, Mai Har Sham

**Affiliations:** 1 School of Biomedical Sciences, Li Ka Shing Faculty of Medicine, The University of Hong Kong, Pokfulam, Hong Kong SAR, China; 2 Department of Obstetrics and Gynaecology, The Chinese University of Hong Kong, Hong Kong, China; 3 Department of Ophthalmology and Visual Sciences, The Chinese University of Hong Kong, Hong Kong, China; 4 Centre for Reproduction Development and Growth, Li Ka Shing Faculty of Medicine, Pokfulam, Hong Kong SAR, China; Purdue University, UNITED STATES

## Abstract

Cataract is the most common cause of visual loss in humans. A spontaneously occurred, autosomal dominant mouse mutant *Secc*, which displayed combined features of small eye, cataract and closed eyelid was discovered in our laboratory. In this study, we identified the mutation and characterized the cataract phenotype of this novel *Secc* mutant. The *Secc* mutant mice have eyelids that remain half-closed throughout their life. The mutant lens has a significant reduction in size and with opaque spots clustered in the centre. Histological analysis showed that in the core region of the mutant lens, the fiber cells were disorganized and clefts and vacuoles were observed. The cataract phenotype was evident from new born stage. We identified the *Secc* mutation by linkage analysis using whole genome microsatellite markers and SNP markers. The *Secc* locus was mapped at chromosome 1 flanked by SNPs *rs3158129* and *rs13475900*. Based on the chromosomal position, the candidate cataract locus γ-crystallin gene cluster (*Cryg*) was investigated by sequencing. A single base deletion (299delG) in exon 3 of *Cryga* which led to a frame-shift of amino acid sequence from position 91 was identified. As a result of this mutation, the sequences of the 3^rd^ and 4^th^ Greek-key motifs of the γA-crystallin are replaced with an unrelated C-terminal peptide of 75 residues long. Coincidentally, the point mutation generated a *HindIII* restriction site, allowing the identification of the *Cryga*^*Secc*^ mutant allele by RFLP. Western blot analysis of 3-week old lenses showed that the expression of γ-crystallins was reduced in the *Cryga*^*Secc*^ mutant. Furthermore, in cell transfection assays using *Cryga*^*Secc*^ mutant cDNA expression constructs in 293T, COS-7 and human lens epithelial B3 cell lines, the mutant γA-crystallins were enriched in the insoluble fractions and appeared as insoluble aggregates in the transfected cells. In conclusion, we have demonstrated that the *Secc* mutation leads to the generation of Cryga^Secc^ proteins with reduced solubility and prone to form aggregates within lens cells. Accumulation of mutant proteins in the lens fibers would lead to cataract formation in the *Secc* mutant.

## Introduction

Cataract is the opacification of the lens, it is the most common cause of visual loss in humans. Congenital cataract has an estimated incidence of 1–6 per 10,000 births and it is the leading cause of visual disability in children worldwide [1–3]. To date, about 15 cataract-related genes have been identified from patients with inherited cataract, these include: (a) genes encoding the structural proteins crystallins: *CRYAA*, *CRYAB*, *CRYBA1*, *CRYBB1*, *CRYBB2*, *CRYGC*, and *CRYGD*; (b) genes encoding the membrane proteins of the lens: *MIP*, *LIM2*, *GJA1*, *GJA3* and *GJA8* (codes for MIP26, MP19, connexin43, connexin46 and connexin50 respectively); (c) genes encoding cytoskeletal proteins, e.g. *BFSP2;* and (d) transcription factor genes *PITX3* and *HSF4*. However, even identical mutations can give rise to different clinical phenotypes; hence environmental or other genetic modifiers may contribute to the phenotype of a particular individual [2,3].

Opacification of the lens is a well recognized phenotype in mice, many mouse cataract mutant alleles that occur spontaneously or identified from mutagenic screens have been documented to date [1,4], contributing to an expanded list of candidate genes for human cataract. Most of the mutant alleles are mapped to the crystallin gene families but there remain a number of cataract mutants with unknown genetic basis. The advantage of cataract mouse mutants is the availability of series of mutant alleles which greatly facilitate the studies of genotype-phenotype correlations and the understanding of the roles of these genes both in normal lens development and in cataract formation.

Many mutations causing cataract in mice also affect the overall growth of the eye, leading to an associated small eye phenotype. Examples of these mouse mutants include genes responsible for synthesizing structural proteins and gap junction proteins such as *Cryaa* [5], *Cryge*^*Cat2-t*^, *Gja1* [6]; and other mutant alleles with unknown mutations such as *ldis1* [7], *Cat3*^*vao*^ [8] and *Tcm* [9]. There are also genes that lead to combined cataract and microphthalmia phenotypes in human patients, *MAF* [1] and *CHX10* [10], but mutations of these same genes in mice cause different ocular abnormalities [1, 11]. Also, there are a number of microphthalmia mutants that do not develop cataract, e.g. *Mab21l1* [12], *Mitf*. All these mutant studies suggest a coordinated development of the lens and other tissues of the eye, but the cellular and molecular link for their development is not well understood. There are a few mouse mutants that have closed eyelid, e.g. *Cle*, *Bmp* [13], but the correlation between the mutation and the phenotype remains unknown.

We have identified a novel mouse mutant named *Secc* which displays small eye, cataract and closed eyelid. The abnormal ocular phenotypes are different from mutants described in the literature and mouse genome database. The *Secc* mutant founder was originally identified among a colony of *FVB/NJ* inbred mice used for our routine transgenic DNA microinjection experiments. However, genotyping using transgene specific primers and probes could not detect any transgene DNA in the mutant genome, suggesting that the phenotypes came from a *de novo* mutation unrelated to transgene insertional mutagenesis. In this study, by gene mapping and direct candidate gene sequencing, we identified the causative mutation of the *Secc* mutant to be located on the γA-crystalline gene *Cryga*. We have shown by protein and cell transfection analyses that the mutant protein formed aggregates with reduced solubility, perturbing normal cell maintenance and leading to cataract formation.

## Materials and Methods

### Animals and Breeding

A spontaneous mutant mouse (named *Secc*) with small eye, closed eyelid and cataract was discovered among a colony of *FVB/NJ* mice. The original stock of *FVB/NJ* and *C57BL/6J* were obtained from the Laboratory Animal Unit of the University of Hong Kong. In a backcross breeding scheme, *Secc* mutant inbred strain in *FVB/NJ* background was first outcrossed with wild type *C57BL/6J* to generate F1 heterozygous mutant mice. Then, F1 mutants were backcrossed with wild type *C57BL/6J* mice to obtain N2 and N3 generations [14]. All mutant mice were observed to be fully viable and fertile. Animal experiments in this study were approved by animal research ethics committee of the University of Hong Kong (CULATR No. 1184–05).

### Lens Morphology and Histological Analysis

For gross phenotype analysis, lenses were prepared under a dissecting microscope (Leica MZ8), images were captured with a Sony DXCS500 digital camera on a Leica FL III microscope. For histological analysis, mouse eyes were dissected and fixed in 4% paraformaldehyde, 7μm sections prepared and stained with hematoxylin and eosin, examined with an Olympus BX51 microscope and images captured with an Olympus DP72 camera.

### Genetic Mapping

DNA samples used for genotyping were extracted from backcrossed mice of the N2 and N3 generations as described above. Microsatellite markers (81 in total) which were specific for the *FVB/NJ* and *C57BL/6J* mouse strains and spaced on average about 20cM across all the autosomal chromosomes were selected and obtained commercially (Applied Biosystems). The microsatellite markers were amplified by PCR and scored by the ABIPRISM 3700 DNA sequencer (Size standard, GS500 (-250) LIZ). The ratio of parental allele for each marker, reflected by heterozygosity percentage, was calculated and evaluated by Chi-square test for significant linkage.

Informative single nucleotide polymorphisms (SNPs) were obtained by searching the Mouse Genome Informatics (MGI) database (http://www.informatics.jax.org). A total of 35 SNPs on chromosome 1, which can differentiate the *FVB/NJ* and *C57BL/6J* mouse strains, were used for the mapping experiment. The genotyping procedures were carried out using a high throughput SEQUENOM MassARRAY system. A region covering the selected SNP was amplified by PCR using specific primers. The PCR products containing the specific SNPs were extended using oligonucleotide probes (hME primers) with deoxynucleotides and dideoxynucleotides so as to produce extended products with different size and mass for homogeneous MassEXTEND (hME) assay. The mass of each extended product was measured and analyzed using SEQUENOM MALDI-TOF mass spectrometry. All oligonucleotides used in both the PCR and extension were designed by the MassARRAY software.

### Gene Sequencing

By linkage analysis, we mapped the *Secc* critical region containing the *Cryg* gene cluster (*Cryga*-*Crygf*). All exons and splicing regions of the *Cryg* gene cluster from wildtype and homozygous mutant genomic DNAs were amplified and sequenced with specific primers using BigDye terminator v3.1 cycle sequencing kit (Applied Biosystems), then further analyzed with the ABIPRISM 3700 DNA sequencer. Sequence results were compared to reference sequences from the NCBI (GeneBank NM_007774, Cryga; NM_144761, Crygb; NM_007775, Crygc; NM_007776, Crygd; NM_007777, Cryge; NM_027010, Crygf).

### Lens Protein Extraction and Western Blot

Fresh lenses were dissected from the eyes of postnatal day 21 (P21) mice. Lens samples (2 pairs) from wild type (50ul per pair of lens) and mutant (25ul per pair of lens) mice were homogenized in radioimmunoprecipitation (RIPA) lysis buffer (Upstate, Temecula, CA) containing 0.5M Tris-HCl, 1.5M NaCl, 2.5% deoxycholic acid, 10% Nonidet P-40, 10mM EDTA and protease inhibitor cocktail (Complete, Roche) with sonication. The concentrations of the protein extracts were determined. Protein samples were denatured in SDS buffer and equal amount of protein extracts (5μg) were loaded onto a 16% SDS-PAGE gel for separation and detection by western blot using antibodies against γ-crystallin (Santa Cruz; SC-27746; 1:100) and β-actin (Sigma; A5441; 1:3000). The analyses were performed in triplicate.

### Generation of Myc-Tagged γA-Crystallin DNA Constructs

Total RNA from wildtype and homozygous mutant lens were isolated using Trizol reagent (Invitrogen). cDNA was synthesized by RT-PCR using oligo-dT primer and SuperScript III reverse transcriptase (Invitrogen). Full length, partial and *Secc Cryga* cDNAs were amplified using specific primers and cloned into the EcoR1/ EcoRV sites of the plamid pCMV-Tag 3B (Stratagene) to generate myc-Cryga^WT^, myc-Cryga^*Secc*^ and myc-Cryga^Partial^ expression constructs. DNA sequence of the constructs were verified by sequencing.

### Cell Culture and Transfection

Human Lens Epithelial (HLE) cell line B3, 293T and COS-7 cell lines were maintained in Dulbecco modified Eagle medium (DMEM) supplemented with 10% fetal bovine serum (FBS) and 1% antibiotics and incubated at 37°C in a humidified chamber with 5% CO_2_ balanced with air. The absence of endogenous γ-crystallins was verified by RT-PCR before transfection. Cells were transfected with myc-Cryga^WT^, myc-Cryga^*Secc*^ or myc-Cryga^Partial^ expression contructs using FuGene 6 (Fugene) according to the manufacturer’s protocol. To monitor the transfection efficiency, the pEGFP-N1 expression vector (Clontech) was co-transfected as internal control.

### Analysis of Solubility of γA-Crystallin

Cells expressing WT or mutant myc-tagged Cryga were washed twice with ice-cold PBS, lysed in RIPA buffer (5 x 10^6^ cells/ml) for 30 minutes on ice and then sonicated. After centrifugation, the supernatant was collected as RIPA-soluble fraction. The pellet containing RIPA-insoluble proteins was washed twice with ice-cold PBS, sonicated and denatured in 9M Urea-RIPA buffer (5 x 10^6^ cells/ ml). To obtain total protein extract, transfected cells (5 x 10^6^ cells/ml) were lysed in 9M Urea-RIPA buffer and sonicated.

Total protein, RIPA-soluble and RIPA-insoluble proteins from samples equivalent to 7.5 x 10^5^ cells were analyzed by 16% SDS-PAGE and Western blotting using antibodies against c-Myc (Santa Cruz; SC-789; 1:1000), GFP (Abcam; ab6556; 1:2000), β-actin (Sigma; A5441; 1:3000) and appropriate HRP-conjugated secondary antibodies (anti-rabbit 656120, anti-mouse 626520; 1:8000). Signals were detected by ECL (GE Healthcare). Immunostaining of GFP and β-actin served as loading controls for transfected cells and protein amounts respectively.

## Results

### Morphologies of the *Secc* Mouse Mutant

**Fig 1:**
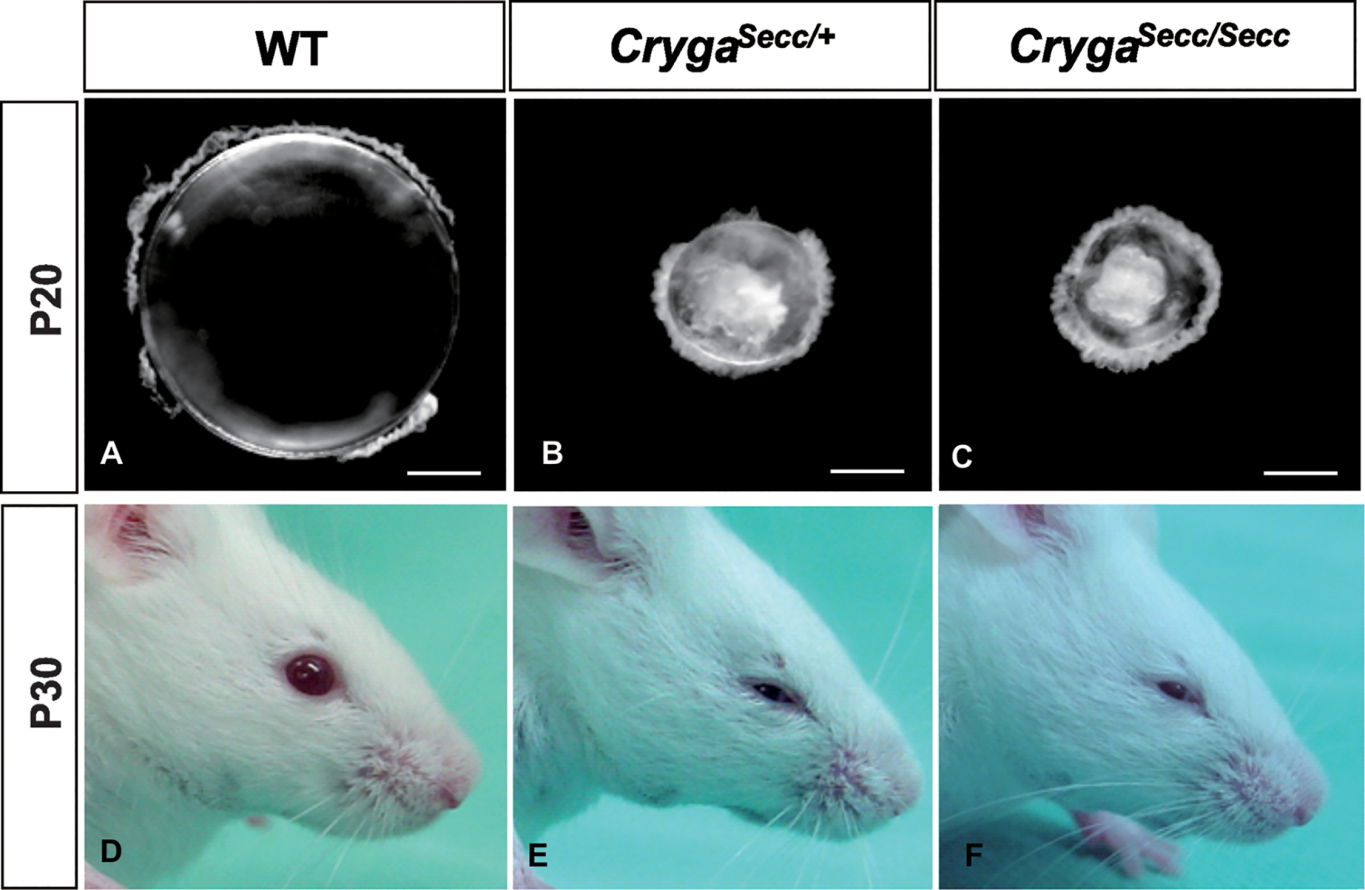
Gross abnormalities of the *Secc* mutant mouse eye. Wildtype FVB/NJ (A, D), heterozygous (B, E) and homozygous (C, F) *Secc* mutant lenses and mice at P20 and P30. *Secc* mutant mice have closed eyelid (E, F), smaller eyes (B, C, E, F) and lens opacity (B, C). Scale bars: 500μm (A-C).

We have identified a spontaneously occurring mouse mutant named *Secc*, which displays combined features of small eye, cataract and closed eyelid. The transmission of the abnormal eye phenotype is consistent with a single gene autosomal dominant mode of inheritance. The eye phenotypes of heterozygous and homozygous mutants are similar. The *Secc* mutants do not fully open their eyelids which remain half-closed throughout life (Fig 1E and 1F), they do not display any eye blinking reflex when tested with light or approaching object. Dissection of mutant eyes and lenses revealed an obvious and significant reduction in their size; opaque spots clustering around the centre of the lens could be readily recognized (Fig 1B and 1C).

### Congenital Cataract Developed in *Secc* Mutant Lenses

**Fig 2:**
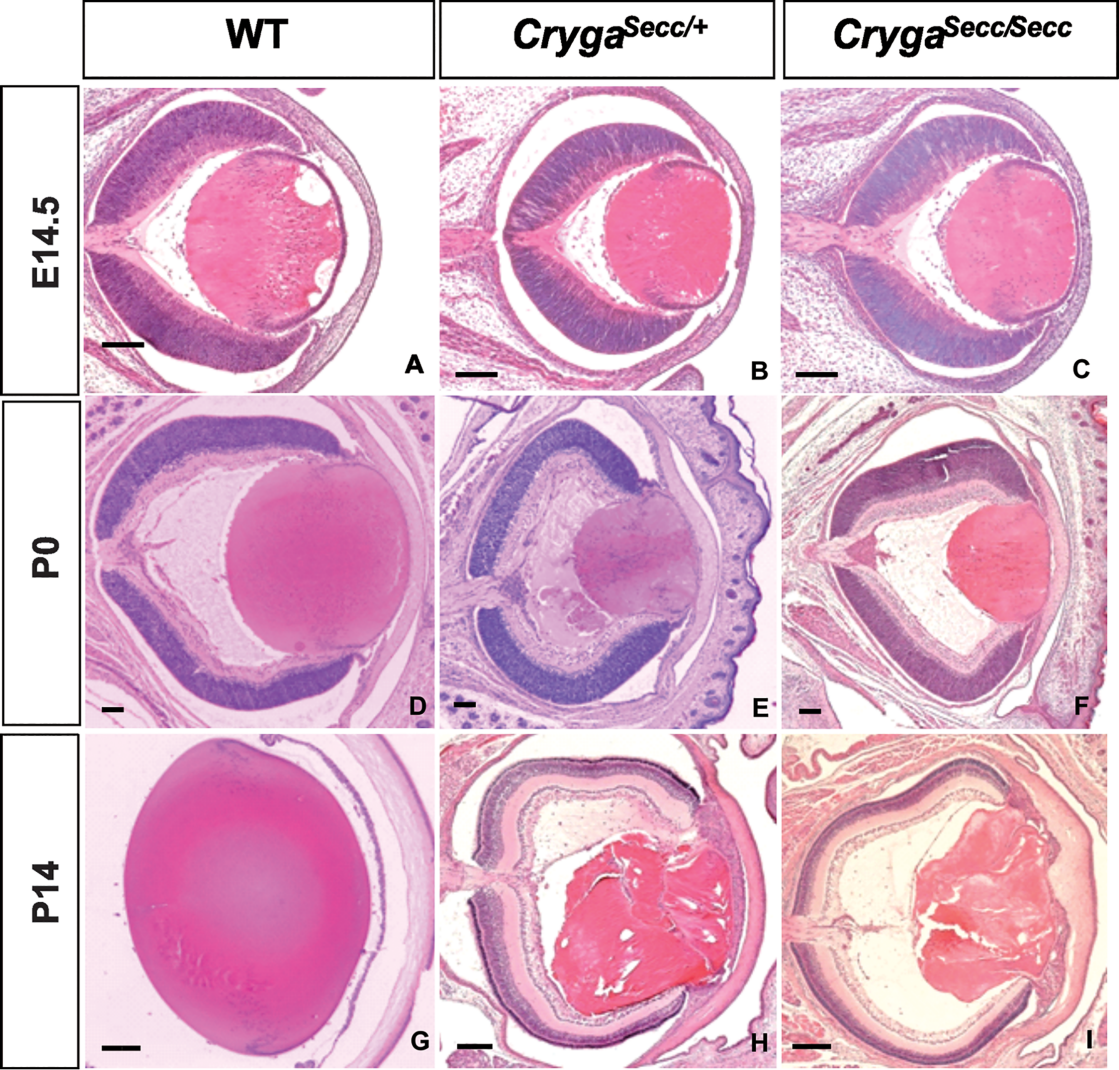
Histological analysis of *Secc* mutant eye. Lens abnormalities were first observed at embryonic E14.5 (B & C) mutant eyes. The lens defects progressed and became more severe by neonatal stages P0 (E & F) and P14 (H & I) as compared with wildtype (A, D, G). Scale bars: 100μm (A-F) and 200μm (G-I).

By histological analysis we found that abnormal lens phenotypes could be observed as early as E14.5 in the *Secc* mutants (Fig 2B and 2C). In the core region of the mutant lenses, the fiber cells were disorganized and swollen, clefts and vacuoles were observed in the cortex of the mutant lenses. These phenotypes became more severe in new born P0 mutant mice (Fig 2E and 2F). In wildtype postnatal P14 lens, fiber cell nuclei could only be seen in the lens cortex region with but not in the lens core (Fig 2G). However, in P14 mutants, nucleus-like structures could be readily observed in the centre of the lens, the morphology of the fiber cells and structure of the lens were disorganized (Fig 2H and 2I). No significant difference could be observed between heterozygous and homozygous mutants.

### Linkage Analysis Using Strain-Specific Genetic Markers

The *Secc* mutant arose among a colony of FVB/NJ inbred mice. To identify the mutation, we generated hybrid congenic mouse strain to transfer the mutant allele to the C57BL/6J genetic background [14]. We set up outcross and then backcross between the *Secc* mutant and wildtype C57BL/6J to obtain N2 hybrids and performed linkage analysis. Eighty-one microsatellite markers specific for the two mouse strains and spaced on average about 20cM across all the autosomal chromosomes were selected. *Secc* mutants from the N2 generation were genotyped using these markers. The ratio of parental allele for each marker, reflected by heterozygosity percentage, was calculated and evaluated by chi-square test for significant linkage. As shown in Table 1, a high probability of linkage between the Chromosome 1 markers D1Mit169, D1Mit132, D1Mit215, D1Mit440 and the *Secc* mutant locus was observed.

**Fig 3:**
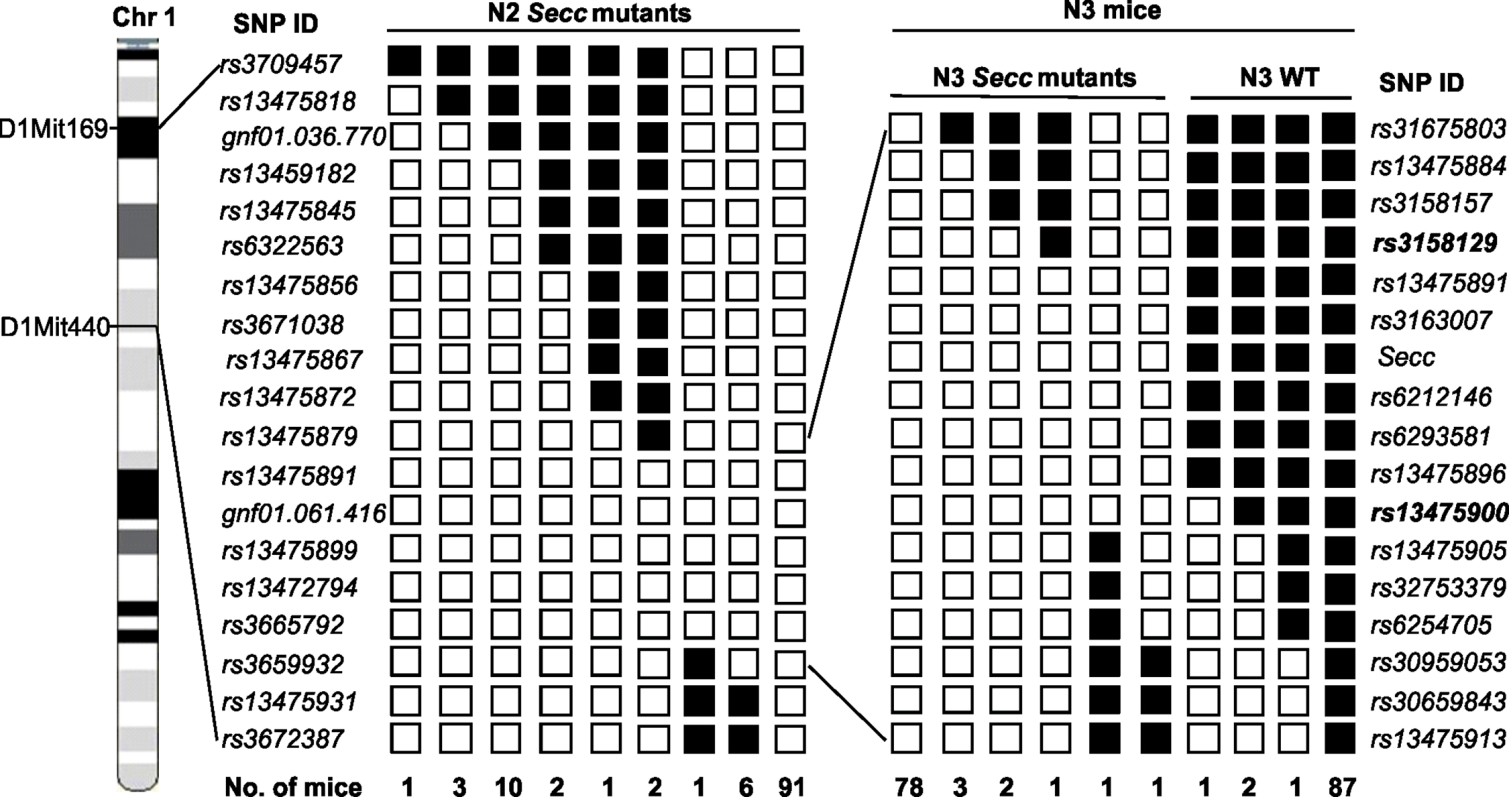
Haplotype analysis of SNP genotypes. A total of 117 N2 *Secc* mutants were genotyped using 19 SNP markers. The critical region spanning the *Secc* locus is mapped between the markers *rs13475879* (57.8Mb) and *rs3659932* (69.2Mb) within a range of around 11Mb. Further mapping was performed by genotyping 177 N3 mice with additional 16 SNP markers covering the region. By defining flanking proximal and distal recombinants, the *Secc* locus is mapped between the markers *rs3158129* (61.8Mb) and *rs13475900* (65.9Mb) spanning around 4.1Mb. Clear boxes represent the FVB/NJ alleles, and black boxes the C57BL/6J alleles. The number of offspring inheriting each haplotype of chromosome is given at the bottom of each column.

Next, we selected 19 strain-specific SNP markers spaced approximately 1.5Mb apart and evenly spanning across the susceptible intervals D1Mit169 –D1Mit440 on Chromosome 1, for further mapping of the mutant locus. Genotyping was performed on 117 *Secc* mutants from the N2 generation. The *Secc* locus was mapped to an 11Mb region in Chromosome 1 which was flanked by *rs13475879* (57.8Mb) and *rs3659932* (69.2Mb) (Fig 3, left panel). Further refined mapping was performed using another 16 strain-specific SNP markers distributed across the mapped 11Mb region. A total of 177 N3 mice, including 91 wildtype and 89 mutants, were genotyped. By defining the flanking proximal and distal recombinants, the *Secc* locus was mapped between the markers *rs3158129* (61.8Mb) and *rs13475900* (65.9Mb), spanning around 4.1Mb (Fig 3, right panel).

**Table 1:** Summary of linkage analysis with Chromosome 1 microsatellite markers.

Microsatellite marker	Position (cM)	Heterozygosity	*p* value	LOD score
**D1Mit430**	10	71%	5.61 x 10^−4^	2.59
**D1Mit169**	15	82%	2.23 x 10^−6^	4.85
**D1Mit132**	43.1	93%	2.29 x 10^−10^	8.74
**D1Mit215**	47	91%	4.94 x 10^−11^	9.39
**D1Mit440**	54	87%	3.17 x 10^−8^	6.65
**D1Mit60**	58.7	78%	2.87 x 10^−6^	3.80
**D1Mit495**	67	80%	8.70 x 10^−7^	5.26
**D1Mit102**	73	72%	1.07 x 10^−3^	2.33
**D1Mit507**	87.8	68%	3.18 x 10^−3^	1.89
**D1Mit292**	107.3	64%	6.93 x 10^−2^	0.72

### Mutation Identification by Direct Sequencing

**Fig 4:**
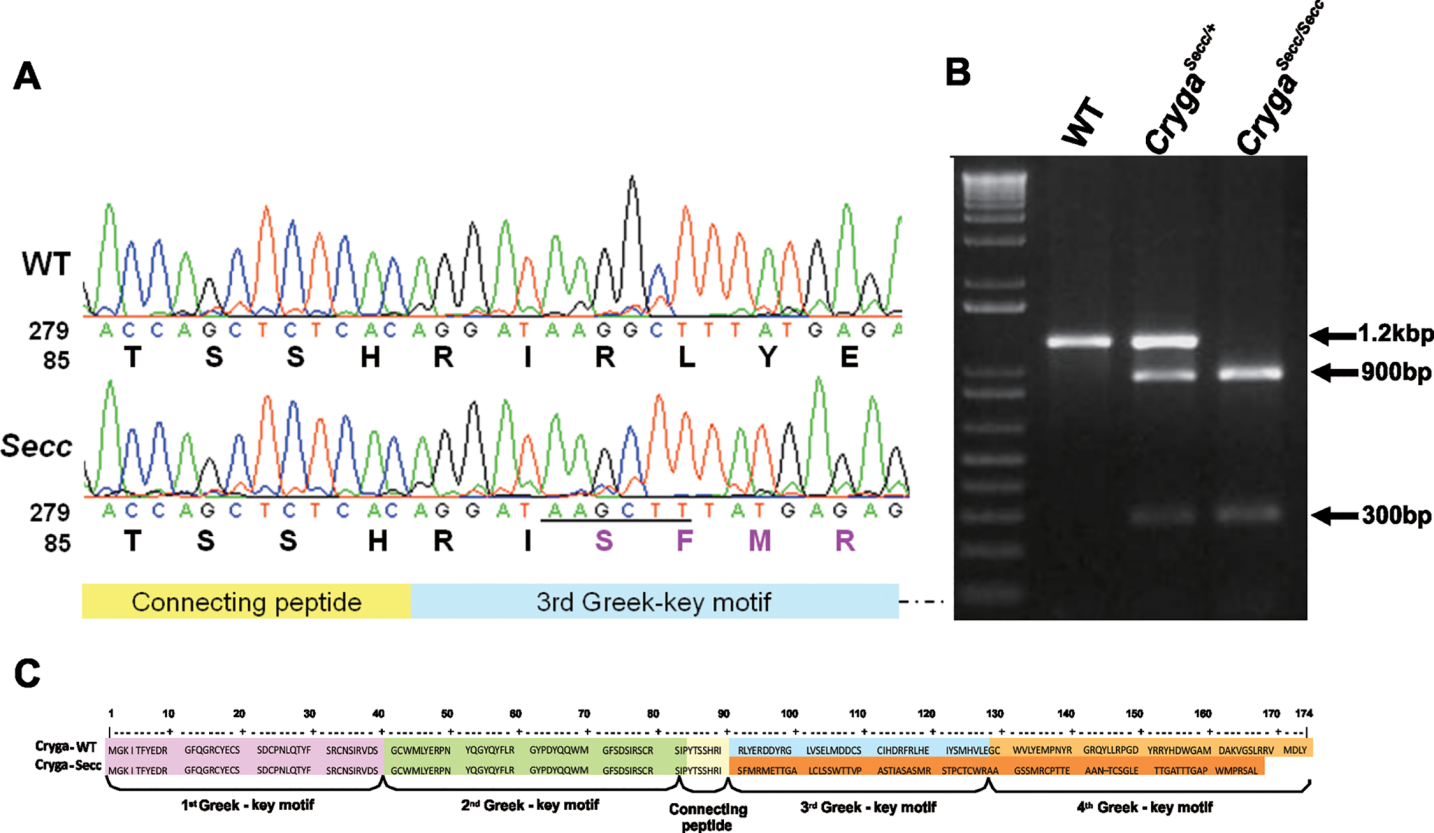
Identification of the *Secc* mutation in the *Cryga* gene. (A) DNA sequencing revealed a single base (G) deletion in the exon 3 of *Cryga* gene, resulting in a frame-shift of amino acid codes from position 91. The mutation also generated a *HindIII* restriction site (AAGCTT, underlined). (B) RFLP (restriction fragment length polymorphism) analysis of the *Secc* mutation by PCR and *HindIII* digestion. The region spanning *Cryga* exon 3 from wildtype and mutant were amplified, digested with *HindIII*, and examined in a 1.5% agarose gel. (C) Schematic alignment of the wild-type and *Secc* mutant Cryga amino acid sequences. The position of the four Greek key motifs and connecting peptide of γA-crystallin are indicated.

Within the *Secc* critical region in the 4.1Mb interval identified by linkage analysis, the candidate *Cryg* gene cluster is located, prompted us to examine potential mutation in this gene cluster by direct sequencing. Using homozygous *Secc* mutant genomic DNA, we sequenced the exons and splice junctions of all 6 *Cryg* genes and identified a single G deletion at nucleotide position 299 in exon 3 of *Cryga* (Fig 4A). No additional coding mutation was detected in the other five members of the cluster (*Crygb-Crygf*). The single G deletion generated a *HindIII* restriction site (Fig 4A underlined sequence), therefore we could confirm the *Secc* mutation by restriction fragment length polymorphism (RFLP). As shown in Fig 4B, *HindIII* digestion of a 1.2kb fragment containing the *Secc* mutation site produced 2 bands (300 and 900bp) in homozygous mutant DNA, while heterozygous mutant DNA displayed a full-length band from the wildtype allele, and 2 bands from the *Secc* mutant allele. The *Secc* mutation would cause a frameshift at the 91^st^ amino acid and disrupt the 3^rd^ and 4^th^ Greek-key motifs, producing a mutant Cryga^*Secc*^ protein with an unrelated peptide of 75 amino acids at the C-terminal (Fig 4C).

### Secc Mutant Lens Protein Cryga^Secc^ Displays Reduced Solubility

**Fig 5:**
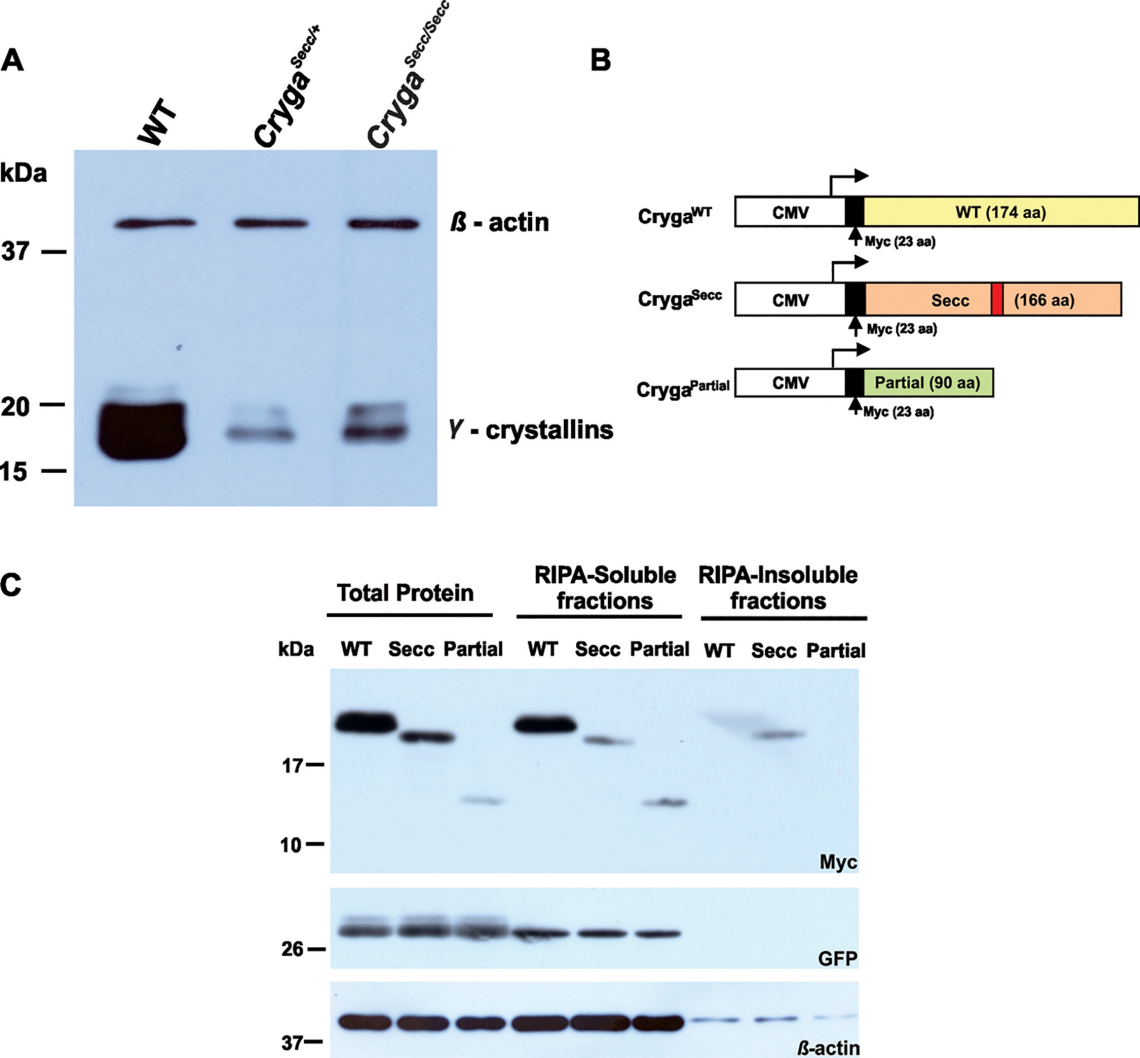
Expression of wildtype and *Secc* mutant γ-crystallins. (A) P21 lenses were analyzed by western blotting with antibodies against total γ-crystallins and β-actin. The expression of γ-crystallins reduced significantly in *Secc* mutant lenses. (B) A schematic diagram of the structure of the wildtypeand mutant myc-tagged Cryga cDNA expression constructs. (C) Analysis of myc-tagged wildtype, *Secc* and partial γA-crystallin expression in COS-7 cells. Immunostaining of GFP and β–actin indicated the transfection efficiency and protein loading in each of the samples.

To investigate the effect of the *Secc* mutation on the expression of the protein, western blot analysis was performed. The predicted mutant protein is estimated to have a molecular mass of approximately 18.7kDa, which is smaller than all other γ-crystallins. However, analysis of soluble proteins extracted from P21 mutant lenses using a pan-γ-crystallin antibody failed to show any protein band of the expected size (18.7kDa). Moreover, the expression levels of total γ-crystallins in the mutant lenses were greatly reduced with reference to the β-actin protein (Fig 5A). As aggregation of crystallin proteins has been described in cataractogenesis[15,16], we hypothesized that the Cryga^Secc^ mutant protein might form aggregates and could not be recovered from the lens protein extracts using standard procedures.

To test our hypothesis, we characterized the expression of the Cryga^Secc^ mutant protein in a cellular model. Myc-tagged cDNA expression vectors for full-length Cryga^WT^ and Cryga^Secc^ mutation were generated. We also constructed a Cryga^Partial^ cDNA with a partial sequence encoding the N-terminal 90 amino acids of γA-crystallin, without the unrelated C-terminal peptide present in the *Secc* mutant protein (Fig 5B). The expression vectors were transfected into COS-7 cells, proteins were collected as total protein as well as soluble and insoluble fractions by using buffers with different reducing power. The expected size of the recombinant proteins are approximately 23kDa for myc-Cryga^WT^, 21kDa for myc-Cryga^*Secc*^ and 13kDa for myc-Cryga^Partial^. Western blot analysis using anti-myc antibody showed that myc-Cryga^WT^ and myc-Cryga^Partial^ were well dissolved in RIPA buffer and could not be detected in the insoluble fraction (Fig 5C). However, considerable amount of myc-Cryga^*Secc*^ was detected in the insoluble fraction, which was recovered using 9M Urea-RIPA buffer (Fig 5C, insoluble fraction). Our results indicated that the Cryga^Secc^ mutant protein indeed had a reduced solubility in standard conditions and could be prone to form insoluble aggregates.

### Mutant Cryga^Secc^ Protein Forms Intracellular Aggregates

**Fig 6:**
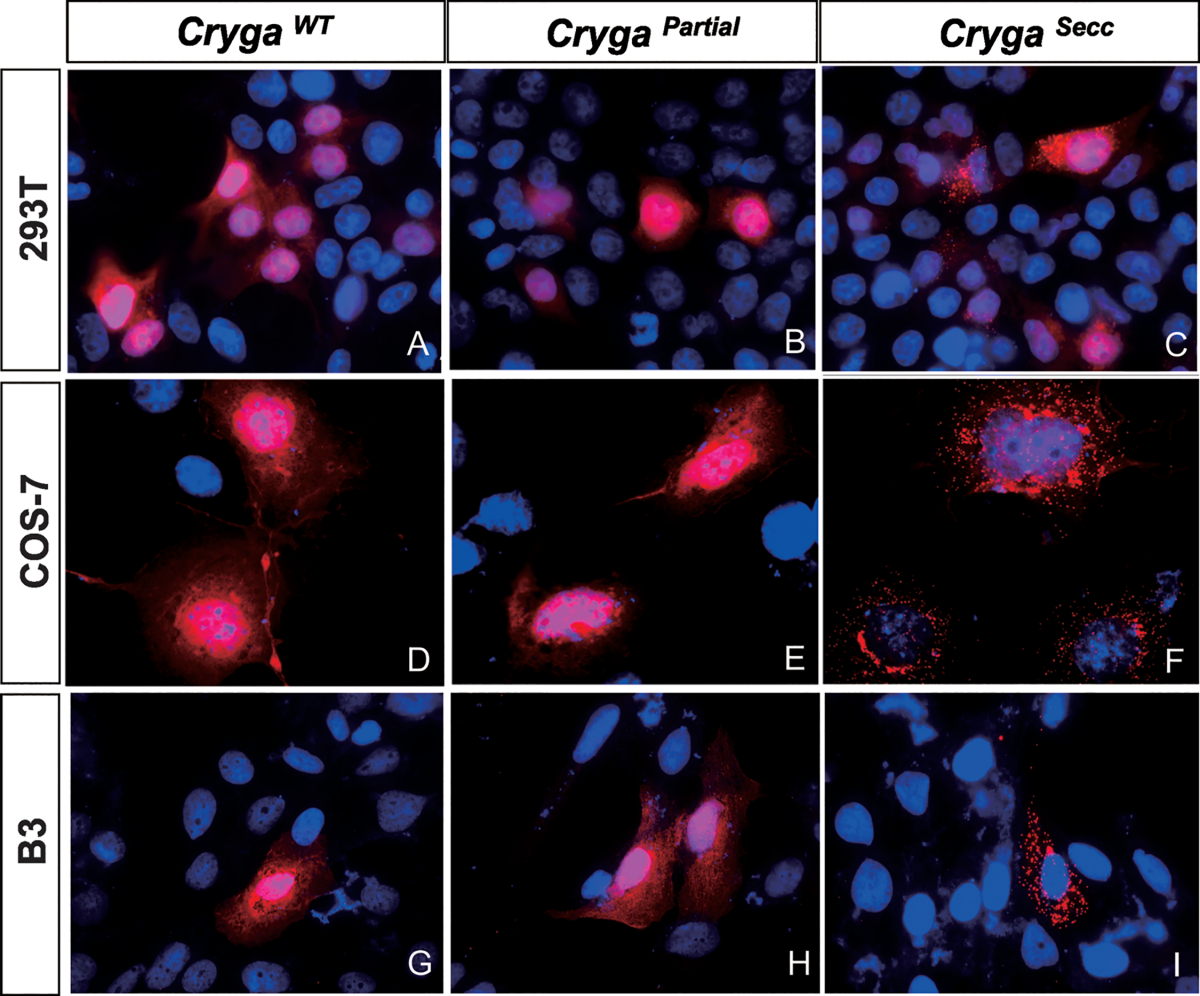
Localization of myc-tagged wildtype, Secc and partial γA-crystallin in 293T, COS-7 and B3 cells. Immunofluorescence of myc-tagged γA-crystallins (red) showing the distribution of wildtype Cryga^WT^ (A, D, G) and Cryga^Partial^ (B, E, H) γA-crystallins in both nucleus and cytoplasm of the transfected cells. Punctate distribution of the Cryga^*Secc*^ mutant protein (C, F, I) was readily observed in the transfected cells, suggesting the formation of mutant protein aggregates.

To further characterize the mutant Cryga^Secc^ proteins in mammalian cells, recombinant myc-Cryga proteins were expressed in three different cell lines 293T, COS-7 and human lens epithelial cell line B3. Analysis by immunostaining of transfected cells with anti-myc antibody showed that myc-Cryga^WT^ and myc-Cryg^Partial^ were localized in both nuclear and cytoplasmic regions (Fig 6), whereas punctate distribution of myc-Cryga^*Secc*^ was observed in all three cell lines examined. Abnormal staining of myc-Cryga^*Secc*^ was mainly found in the cytoplasmic regions (Fig 6C and 6F). Together with the previous observation of the decreased solubility of mutant Cryga^*Secc*^, our results suggested that the mutant Cryga^Secc^ protein could form intracellular aggregates. As no punctate staining could be observed in cells transfected with myc-Cryga^WT^ and myc-Cryg^Partial^ cDNAs, the protein aggregate formation could be due to the presence of the C-terminal unrelated peptide resulted from the frameshift mutation in the *Secc* mutation.

## Discussion

In this study, by linkage analysis and sequencing of candidate genes, we identified a new cataract mutation in the γA-crystallin gene *Cryga*^*Secc*^ in the novel spontaneous mouse mutant *Secc*. The *Secc* mutant was originated in the *FVB/6J* strain, congenital nuclear cataract developed in the mutant strain as early as embryonic day E14.5, shortly after the expression of γ-crystallins was initiated. As demonstrated by our histological analysis (Fig 2), the mutant showed disorganized and swollen fiber cells in the core region of the lenses. As the mice grew, the phenotypes became more severe with clefts and vacuoles formed and rupture of the lens. A regular arrangement of lens fiber cells is essential for maintaining the transparency of the lens. Improper alignment, presence of vacuoles or nuclei within fiber cells, as well as lens rupture will perturb light transmission and cause serious scattering.

The *Secc* mutant mouse strain is a unique cataract model that is different from previously reported γA-crystallin mutants. In addition to the Cryga^*Secc*^ mutation described here, *Cryga*^*1Neu*^ [17] and *Cryga*^*tol*^ [18] which are point mutations with single amino acid substitution in the mouse *Cryga* gene have been previously reported. All three *Cryga* mutations display dominant trait of inheritance and cataract phenotypes. Both *Cryga*^*1Neu*^ and *Cryga*^*tol*^ mutants exhibit nuclear opacities. Dense nuclear opacity, microphthalmia and lens rupture were observed in *Cryga*^*tol*^ mutant at 3 weeks of age. In the *Secc* mutant, lens rupture phenotype occurred at an earlier stage of P14, with the presence of numerous vacuoles. The series of *Cryga* mutants display different extent of phenotype severity in lens defects and microphthalmia, allowing further studies on cataractogenesis and genotype-phenotype correlation. Notably, *Cryga*^*Secc*^ is a frameshift mutation which could lead to the production of a very different mutant γA-crystallin protein and cause a more severe phenotype.

The *Cryga*^*Secc*^ mutant allele has a single nucleotide deletion at n299 and resulted in a frameshift from the 91^st^ amino acid residue. The γA-crystallin protein normally consists of four Greek key motifs, with the 1st and 2^nd^ motif linked to the 3^rd^ and 4^th^ motifs via a short connecting peptide (see Fig 4C). The *Cryga*^*Secc*^ mutation occurred immediately after the connecting peptide region. As a result of the frameshift, the 3^rd^ and 4^th^ Greek key motifs were replaced with an unrelated peptide of 75 amino acids long. The *Cryga*^*Secc*^ mutant gene thus encodes an abnormal protein of 166 amino acids and approximately 18.7kDa. The *Cryga*^*Secc*^ mutation could significantly affect the structure of the mutant γA-crystallin. It is not known whether the C-terminal unrelated peptide has any ordered conformation; and more importantly, whether the folding of the first two Greek-key motifs would be affected in the mutant protein. The conformation of the mutant protein is likely to be different from the wildtype and other reported mutant crystallins. Therefore, protein conformation modulated cataractogenic defects may occur in the *Cryga*^*Secc*^ mutant lens.

It has been suggested that the mechanism of cataract formation is due to the presence of intranuclear and cytoplasmic inclusions involving altered folding of γ-crystallins. Mutant crystallins may exhibit altered protein conformation, stability and protein-protein interactions; these alterations would lead to amyloid and aggregation formation [19–26] and cause cataract in lens. Using an antibody that recognizes members of normal γ-crystallins, we had not been able to detect any mutant Cryga^Secc^ proteins in the mutant lens extracts by western blotting. Moreover, the overall expression level of γ-crystallins in the mutant lenses were reduced (Fig 5A). The mutant Cryga^Secc^ protein probably could not be recognized by the antibody used, or that the mutant protein might be unstable and could not be detected. It is also possible that the Cryga^Secc^ mutant crystallin was not produced. For instance, the mutant mRNA transcripts might be degraded due to nonsense-mediated mRNA decay (NMD) and the mutant protein would not be synthesized. However, we considered the possibility that the mutant γ-crystallin did express, but formed entangled protein aggregates and became insoluble in standard buffer conditions, hence could not be detected.

By cell transfection analysis using recombinant myc-Cryga^WT^, myc-Cryga^*Secc*^ and myc-Cryga^Partial^ cDNAs, we confirmed that significant portion of Cryga^*Secc*^ mutant proteins form insoluble aggregates in different cell lines, including the human lens epithelial cell line B3. Interestingly, the levels of expression of full length and partial mutant proteins were lower than that of wildtype, indicating that the mutant proteins could be less stable than the wildtype. Our results suggest that the *Secc* mutation could lead to folding defects of the mutant γA-crystallin protein which resulted in their aggregation within cells.

It is believed that γ-crystallins are monomeric proteins interacting not only among themselves, but also with other proteins such as α-crystallin [27, 28], vimentin [29] and aquaporin [30, 31]. Therefore, aggregation of mutant γ-crystallins could lead to reduction of other crystallins, as a result of abnormal protein interactions and non-specific aggregate formation. The formation of these mutant aggregates might lead to other downstream effects, such as denucleation process of lens fiber cells and down-regulation of other γ-crystallin members, contributing to the cataractogensis process.

In mouse lens ablation study using crystallin promoter driven expression of toxic gene constructs, phenotypes were not only restricted to the lens but also in cornea, iris and ciliary body [32]. These studies support the hypothesis that the lens provides the source of signals for the anterior part of the eye and retina development. In the case of *Secc* mutant, the small eye phenotype may also be due to diminished support from the lens, although no obvious structural abnormality is found in other ocular tissues. Interestingly, the *Secc* mutants also display a closed eyelid phenotype, similar to blepharophimosis in human patients. Using a mouse model of tissue specific mutation of the *Foxl2* gene which recapitulated the phenotypes of the human blepharophimosis, ptosis and epicanthus inversus syndrome (BPES), it has been shown that defective development of cranial neural crest and mesodermal cell derived muscles and skeletal components would prevent eyelid closure [33]. The closed eyelid phenotype of the *Secc* mutant is likely to be secondary to the abnormal lens development, the underlying mechanism needs to be further investigated.
